# Functional Dissection of pri-miR-290~295 in Dgcr8 Knockout Mouse Embryonic Stem Cells

**DOI:** 10.3390/ijms20184345

**Published:** 2019-09-05

**Authors:** Ming Shi, Jing Hao, Xi-Wen Wang, Le-Qi Liao, Huiqing Cao, Yangming Wang

**Affiliations:** 1 Institute of Molecular Medicine, Peking University, 5 Yiheyuan Road, Beijing 100871, China (M.S.) (J.H.) (X.-W.W.); 2 Academy for Advanced Interdisciplinary Studies, Peking University, Beijing 100871, China

**Keywords:** DiGeorge syndrome critical region gene 8, primary microRNA, miR-290~295 cluster, embryonic stem cells, cell cycle, pluripotency, self-renewal, differentiation, Nlrp12

## Abstract

The DiGeorge syndrome critical region gene 8 (Dgcr8) knockout strategy has been widely used to study the function of canonical microRNAs (miRNAs) in vitro and in vivo. However, primary miRNA (pri-miRNA) transcripts are accumulated in Dgcr8 knockout cells due to interrupted processing. Whether abnormally accumulated pri-miRNAs have any function is unknown. Here, using clustered regularly interspaced short palindromic repeats system/CRISPR-associated protein 9 (CRISPR/Cas9), we successfully knocked out the primary microRNA-290~295 (pri-miR-290~295) cluster, the most highly expressed miRNA cluster in mouse embryonic stem cells (ESCs), in Dgcr8 knockout background. We found that the major defects associated with Dgcr8 knockout in mouse ESCs, including higher expression of epithelial-to-mesenchymal transition (EMT) markers, slower proliferation, G1 accumulation, and defects in silencing self-renewal, were not affected by the deletion of pri-miR-290~290 cluster. Interestingly, the transcription of neighboring gene nucleotide-binding oligomerization domain, leucine rich repeat and pyrin domain containing 12(Nlrp12) was upregulated upon the deletion of the pri-miR-290~295 cluster. Together, our results suggested that the major defects in Dgcr8 knockout ESCs were not due to the accumulation of pri-miR-290~295, and the deletion of miRNA genes could affect the transcription of neighboring DNA elements.

## 1. Introduction

The essential roles of microRNAs (miRNAs) in modulating gene expression and various cellular processes have been extensively reported [1]. However, the functions of most miRNAs are still unknown. Although different research tools have been rapidly developed for miRNA functional studies, redundancy among miRNA genes is an unavoidable obstacle to uncover their potential fine-tuning roles [2,3]. We and others have shown that deletion of critical components for a small RNA processing machinery, including Dicer1 ribonuclease III (Dicer1) and DiGeorge syndrome critical region gene 8 (Dgcr8), is an effective approach to block mature miRNA production at transcriptome-wide range [3,4,5,6], therefore circumventing the redundancy issue. Dgcr8 is a relatively more specific member of the microRNA processing pathway compared to Dicer [6,7]. Reintroduction of miRNA mimics into Dgcr8 knockout model has been successfully employed to identify functional miRNAs and to study their regulatory mechanisms [3,8,9,10,11,12,13,14,15]. Moreover, short hairpin RNA vector-mediated stable expression of miRNAs could be achieved in Dgcr8 but not Dicer knockout cells [16].

The biogenesis of miRNAs is sophisticatedly controlled at multiple layers. Primary miRNA (pri-miRNA) transcript recognition and cleavage by DROSHA ribonuclease type III (DROSHA) and DGCR8 is the initial step for miRNA maturation [17,18,19]. It is speculated that the interruption of the pri-miRNA processing may lead to the accumulation of large amounts of the primary transcripts in DGCR8 deficient cells. Although pri-miRNAs are considered transient intermediates during the production of mature miRNAs, several reports claimed that pri- and pre-miRNAs have direct regulatory functions in cognate RNA recognition and gene expression [20,21,22,23]. Whether the excessive pri-miRNA would disturb an elaborate regulatory network of gene expression and thereby contribute to the occurrence of abnormal cellular phenotype in Dgcr8 knockout cells has never been directly evaluated.

Here, we dissected the role of the pri-miR-290~295 cluster in Dgcr8 knockout embryonic stem cells (ESCs). The miR-290~295 cluster was chosen for its high abundance and various important functions in ESCs [8,9,10,11,12,14,24]. Using CRISPR/Cas9 mediated knockout strategy [25,26,27,28,29], we deleted the pri-miR-290~295 cluster in Dgcr8 knockout background. The potential role of pri-miR-290~295 in regulating the cell cycle, pluripotency maintenance, and self-renewal was investigated by comparing Dgcr8/ pri-miR-290~295 double knockout to Dgcr8 knockout ESCs. We showed that the deletion of primary transcripts of the miR-290~295 cluster had no overt effects for these processes. Also, in both wild type and Dgcr8 knockout ESCs, the deletion of pri-miR-290~295 locus led to the upregulation in the transcription of neighboring gene Nlrp12. Therefore, the accumulation of pri-miR-290~295 transcripts was not responsible for major defects observed in Dgcr8 knockout ESCs. However, the deletion of miRNA genes might impact the transcription of neighboring genes through mechanisms independent of miRNA production and targeting.

## 2. Results

### 2.1. Establishment of Dgcr8/Pri-miR-290~295 Double Knockout ESCs 

**Figure 1:**
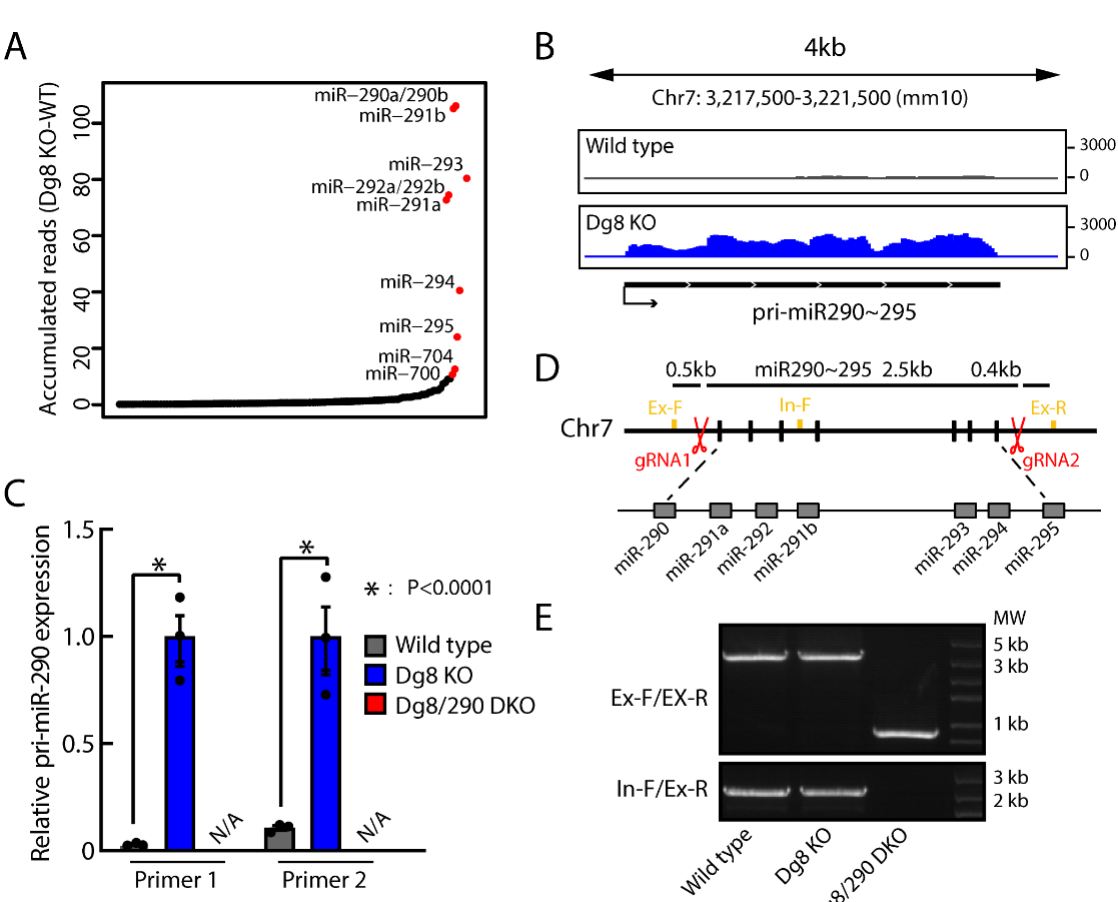
CRISPR/Cas9 mediated pri-miR-290~295 knock out in Dgcr8 knockout embryonic stem cells (ESCs). (**A**) Accumulated reads mapped to pre-miRNA loci. Normalized reads per million total reads are shown. (**B**) RNA-Seq tracks for pri-miR-290~295 locus in wild type and Dgcr8 knockout ESCs. Shown are normalized read counts per million. (**C**) Pri-miR-290~295 levels in ESCs measured by RT-qPCR. Results from two different primer pairs targeting pri-miR-290 were exhibited. Shown are means ± SEM, *n* = 3 independent experiments. The *p*-value was determined by one-way ANOVA followed by two-tailed Tukey’s test. (**D**) A diagram illustrating the genomic locus of the miR-290~295 cluster and the knockout strategy. Locations of the targeting sequences for the gRNAs and of the primers for PCR verification are indicated by red scissors and yellow squares. (**E**) Genomic PCR confirming the deletion of miR-290 fragment using different primers, as shown in Figure 1C. Dg8 KO, Dgcr8 knockout; Dg8/290 DKO, Dgcr8/pri-miR-290~295 double knockout.

RNA-Seq analysis of the wild type and Dgcr8 knockout ESCs showed that pri-miR-290~295 transcripts were significantly accumulated in Dgcr8 knockout ESCs (Figure 1A,B, Table S1). Accumulated reads from miR-290~295 cluster accounted for ~70% of total accumulated reads from all mapped miRNA loci, indicating that pri-miR-290~295 was the major contributor to the pool of accumulated pri-miRNAs in Dgcr8 knockout ESCs. Reverse transcription-quantitative polymerase chain reaction (RT-qPCR) analysis confirmed the accumulation of pri-miR-290~295 in Dgcr8 knockout ESCs (Figure 1C). A previous study [30] has identified an intron between positions +84 and +715 relative to the transcription start site of the miR-290~295 cluster, the splicing of which gives rise to an alternative pri-miR-290~295 transcript. Our RNA-seq analysis confirmed this finding and found that the ratio between spliced versus unspliced transcript was approximately 3 to 1. To determine the impact of pri-miRNAs in Dgcr8 knockout ESCs, we genetically deleted miR-290~295 gene cluster spanning 2.5 kb length of genomic DNA using CRISPR/Cas9 technology (Figure 1D). Knockout of the target fragment was confirmed by genomic PCR analysis (Figure 1E). Besides, the pri-miR-290~295 levels were determined by RT-qPCR using two different sets of primers. As expected, we found that the pri-miR-290~295 transcripts were successfully removed in Dgcr8/pri-miR-290~295 double knockout cells (Figure 1C). These results indicated that pri-miR-290~295 knockout ESCs were successfully established in the Dgcr8 knockout background.

### 2.2. Pluripotency and Epithelial-to-Mesenchymal Transition (EMT)-Like Phenotype in Dgcr8/Pri-miR-290~295 Double Knockout ESCs

**Figure 2:**
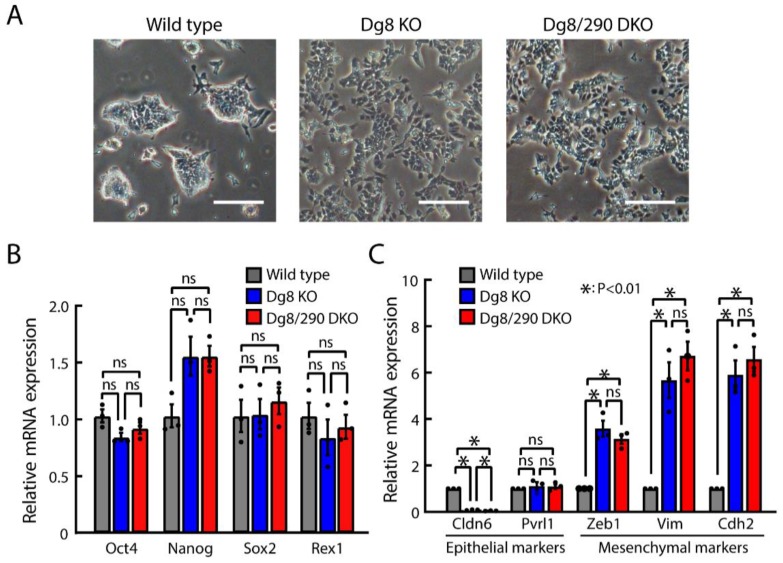
Pri-miR-290~295 transcripts have no role in regulating pluripotency and epithelial-to-mesenchymal transition (EMT)-like property in ESCs. (**A**) Morphology of ESC colonies under standard culture conditions. Representative images from different groups are shown. Scale bars = 200 μm. (**B**,**C**) mRNA levels of pluripotency marker genes (**B**) and EMT-associated genes (**C**) were analyzed by RT-qPCR. The β-actin gene was taken as an internal control. For each gene, data were normalized to the mRNA level of wild type ESCs. Shown are mean ± SEM, *n* = 3 independent experiments. The *p*-value was determined by one-way ANOVA followed by two-tailed Tuke’s test. Dg8 KO, Dgcr8 knockout; Dg8/290 DKO, Dgcr8/pri-miR-290~295 double knockout.

We then determined whether the knockout of pri-miR-290~295 affected the pluripotency state under standard mouse ESC culture conditions. We previously reported mesenchymal-like phenotypes in Dgcr8 knockout ESCs [12]. Here, we found that Dgcr8/pri-miR-290~295 cluster double knockout cells exhibited dispersed and jagged shape (Figure 2A), which is similar to Dgcr8 knockout ESCs. Meanwhile, the wild type ESCs grew into typical tight domed colonies (Figure 2A). These results suggested that Dgcr8/pri-miR-290~295 double knockout ESCs retained mesenchymal-like properties. We then analyzed the mRNA levels of master pluripotency transcription factors, including octamer-binding protein 4 (Oct4), sex determining region Y-Box 2 (Sox2), Nanog Homeobox(Nanog), and reduced expression protein 1 (Rex1), in Dgcr8/pri-miR-290~295 double knockout, Dgcr8 knockout, and wild type ESCs using RT-qPCR, and no significant differences were found among all the groups (Figure 2B), suggesting undisturbed pluripotency maintenance of the Dgcr8/pri-miR-290~295 knockout ESCs. Additionally, EMT-associated markers [31,32], including claudin 6 (Cldn6), poliovirus receptor-related protein 1 (Pvrl1) for epithelium and zinc finger E-box binding homeobox 1 (Zeb1), vimentin (Vim), cadherin 2 (Cdh2) for mesenchyme, were also measured by RT-qPCR. As expected, some of the markers were dysregulated due to DGCR8 deficiency [12]; however, no discrepancy was observed between Dgcr8 knockout and Dgcr8/pri-miR-290~295 double knockout ESCs. These results indicated that the loss of pri-miR-290~295 had no impact on the EMT process. Combined with our previous study [12] on EMT regulation by miRNAs, we concluded that the EMT-like phenotype observed in Dgcr8 knockout ESCs was due to the loss of mature miR-290~295 miRNAs. 

### 2.3. Proliferation and Cell Cycle Progression of Dgcr8/Pri-miR-290-295 Double Knockout ESCs

**Figure 3:**
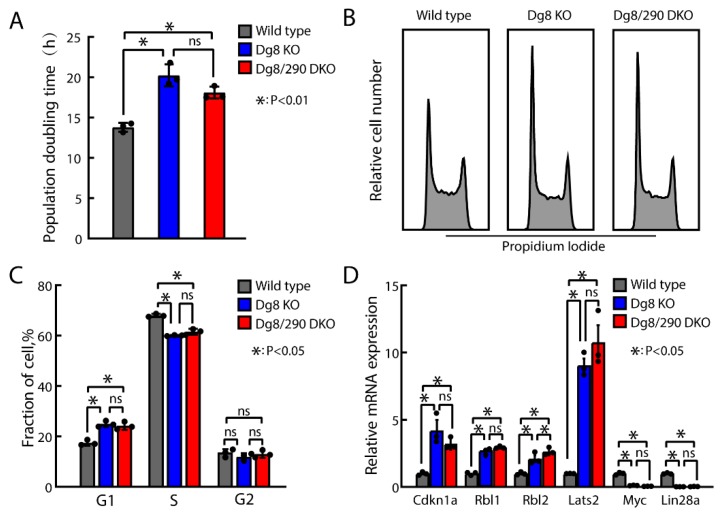
The accumulation of pri-miR-290~295 transcripts has no role in proliferation and cell cycle defects of Dgcr8 knockout ESCs. (**A**) Cell population doubling time was calculated during serial passages in ESC culture. (**B**,**C**) Cell cycle profile with propidium iodide (PI) staining of ESCs was analyzed by flow cytometry, and the fraction of cells in the various phases of the cell cycle was calculated. (**D**) Expression of cell cycle genes in ESCs was determined by RT-qPCR. The β-actin gene was taken as an internal control. For each gene, data were normalized to the mRNA level of wild type ESCs. Shown are mean ± SEM, *n* = 3 independent experiments. The *p*-value was determined by one-way ANOVA followed by two-tailed Tukey’s test. Dg8 KO, Dgcr8 knockout; Dg8/290 DKO, Dgcr8/pri-miR-290~295 double knockout.

Next, we evaluated the effect of pri-miR-290~295 on cell growth. Consistent with our previous results [6,8], Dgcr8 knockout ESCs showed extended population doubling time relative to wild type control (Figure 3A). Besides, the population doubling time of Dgcr8/pri-miR-290~295 double knockout ESCs was similar to that of Dgcr8 knockout ESCs (Figure 3A). Cell cycle analysis demonstrated that compared to wild type control, the Dgcr8/pri-miR-290~295 double knockout ESCs accumulated in the G1 phase with a concomitant decreased fraction in S phase, which is also similar to Dgcr8 knockout cells (Figure 3B,C). Consistently, when compared to wild type ESC control, cell cycle inhibitors, including cyclin-dependent kinase inhibitor 1A (Cdkn1a), RB transcriptional corepressor like 1 (Rbl1), RB transcriptional corepressor like 2 (Rbl2), and large tumor suppressor 2 (Lats2), were similarly upregulated, while proliferation promoting genes lin-28 homolog A (Lin28a) and myelocytomatosis oncogene (Myc) were similarly downregulated in Dgcr8 knockout and Dgcr8/pri-miR-290~295 double knockout ESCs. These data indicated that the accumulation of pri-miR-290~295 transcript had neither beneficial nor detrimental effects on the proliferation of Dgcr8 knockout ESCs.

### 2.4. Embryoid Body Differentiation of Dgcr8/miR-290-295 Double Knockout ESCs

**Figure 4:**
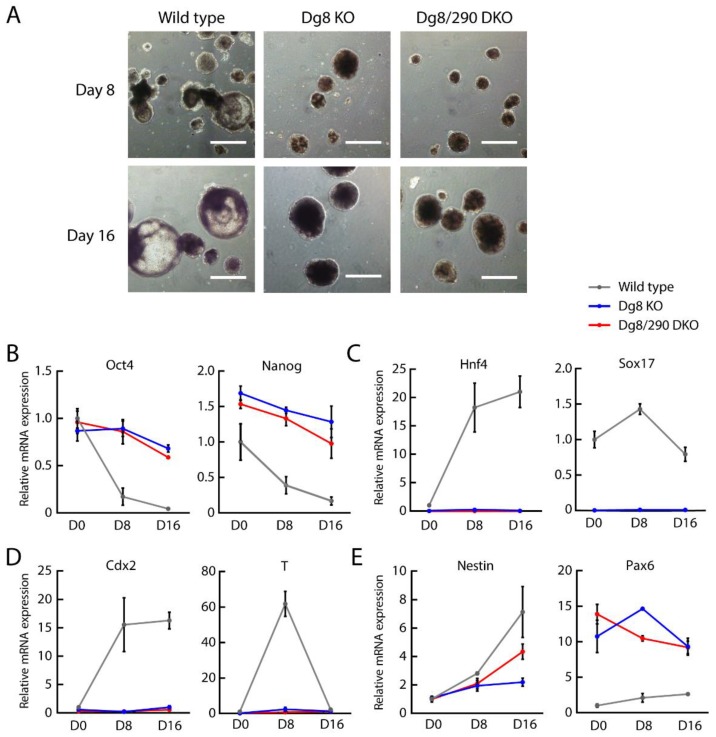
The accumulation of pri-miR-290~295 transcripts has no role for embryoid body (EB) differentiation defects of Dgcr8 knockout ESCs. (**A**) Representative images of embryoid bodies from ESCs with a different background. Scale bars = 500 μm. (**B**–**E**) Expression of pluripotency and differentiation markers at day 0, 8, and 16 of EB differentiation. Expression of representative markers of pluripotency (Oct4, Nanog), endoderm (hepatic nuclear factor 4 alpha (Hnf4), Ssex determining region Y-Box 17 (Sox17)), mesoderm (T-box transcription factor T brachyury (T), caudal type homeobox 2 (Cdx2)), and ectoderm (Pax6, Nestin) was measured by RT-qPCR. The β-actin gene was used as an internal control. For each gene, data were normalized to the mRNA level of wild type ESCs (D0). Shown are mean ± SEM, *n* = 3 independent experiments. The *p*-value was determined by one-way ANOVA followed by two-tailed Tukey’s test. Dg8 KO, Dgcr8 knockout; Dg8/290 DKO, Dgcr8/pri-miR-290~295 double knockout.

To determine the influence of pri-miR-290~295 on ESC differentiation, we induced the formation of embryoid bodies (EBs) by leukemia inhibitory factor (LIF) withdrawal in suspension cultures. While cystic EBs were observed for wild type cells at day 8 and day 16, EBs from both Dgcr8 knockout and Dgcr8/pri-miR-290~295 double knockout cells failed to form a cyst (Figure 4A), suggesting incomplete differentiation. We then measured the expression of marker genes for pluripotency and differentiation by RT-qPCR. As expected [6], Dgcr8 knockout cells failed to silence pluripotency markers as efficiently as wild type cells (Figure 4B). Besides, Dgcr8 knockout cells also failed to upregulate various differentiation markers representing different lineages (Figure 4C–E). A previous study [33] showed that paired box 6 (Pax6) was repressed by mature miRNAs from the miR-290~295 cluster. Our results showed that Pax6 was also upregulated in Dgcr8 knockout ESCs. Importantly, Dgcr8/pri-miR-290~295 double knockout ESCs consistently showed similar results with that of Dgcr8 knockout cells in the expression of pluripotency and differentiation markers (Figure 4B–E). We thereby concluded that the accumulation of pri-miR-290~295 played no role in EB differentiation defects of Dgcr8 knockout ESCs.

### 2.5. Let-7 Mediated Silencing of Self-Renewal in Dgcr8/Pri-miR-290 Double Knockout ESCs

**Figure 5:**
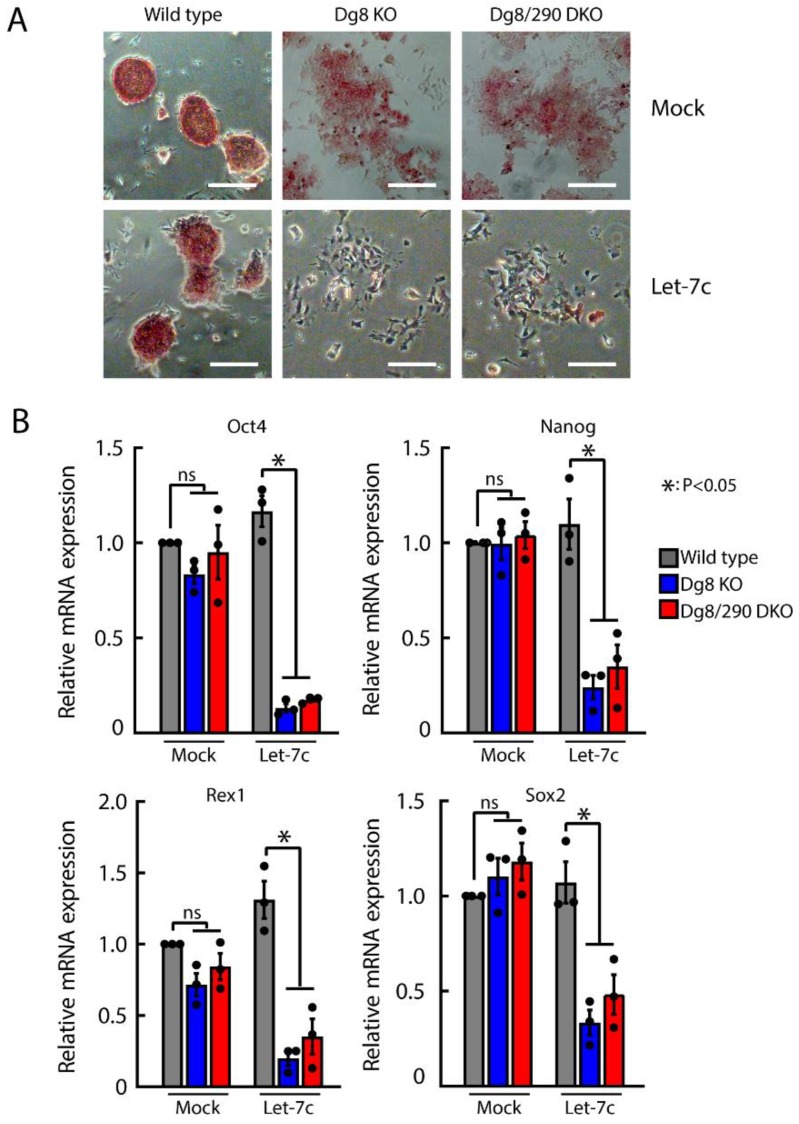
The accumulation of pri-miR-290~295 has no role in let-7 mediated silencing of self-renewal of Dgcr8 knockout ESCs. (**A**) Representative images of ESC colonies with alkaline phosphatase staining after mock or let-7c transfection. Scale bars = 200 μm. (**B**) qRT-PCR analysis of self-renewal genes in ESCs after mock or let-7c transfection. The β-actin gene was used as a control. For each gene, data were normalized to the mRNA level of wild type ESCs. Shown are means ± SEM, *n* = 3 independent experiments. The *p*-value was determined by one-way ANOVA followed by two-tailed Tukey’s test. Dg8 KO, Dgcr8 knockout; Dg8/290 DKO, Dgcr8/pri-miR-290~295 double knockout.

The self-renewal of Dgcr8 knockout but not wild type ESCs can be silenced by let-7c [9,10,12]. To investigate whether the accumulation of pri-miR-290~295 transcripts has any role for the sensitivity of Dgcr8 knockout ESCs to let-7c, we transfected let-7c into Dgcr8 knockout and Dgcr8/pri-miR-290~295 double knockout ESCs, followed by alkaline phosphatase (AP) staining for undifferentiated cells. AP activity remained unaltered upon let-7c treatment in the wild type control, in contrast, markedly reduced signals were observed in both Dgcr8 knockout and Dgcr8/pri-miR-290~295 double knockout ESCs (Figure 5A), indicating loss of their self-renewal abilities. Consistently, pluripotency markers, including Oct4, Nanog, Rex1, and Sox2, were dramatically downregulated by let-7c in Dgcr8 knockout and Dgcr8/pri-miR-290~295 double knockout but not in wild type ESCs (Figure 5B). Together, these results suggested that the accumulation of pri-miR-290~295 transcripts played no role in let-7c mediated silencing of self-renewal in Dgcr8 knockout ESCs.

### 2.6. Impact of Pri-miR-290~295 Knockout on the Transcription of Neighboring Gene

**Figure 6:**
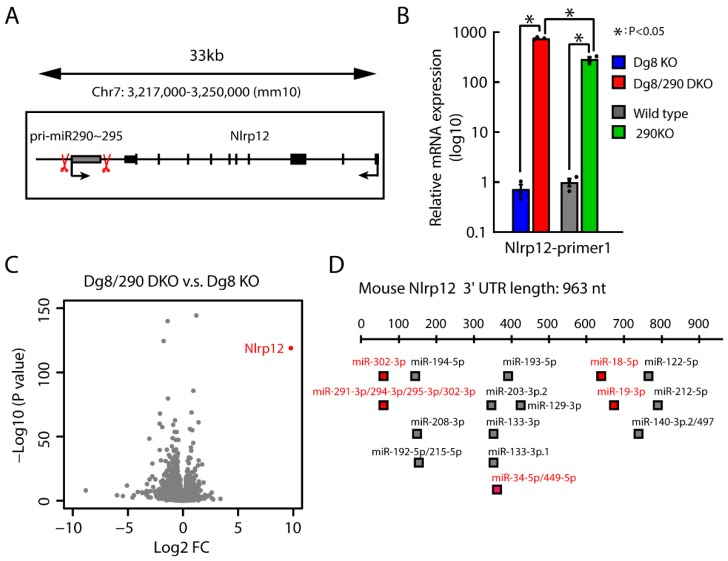
Deletion of pri-miR-290~295 cluster leads to remarkable upregulation of Nlrp12 in both wild type and Dgcr8 knockout ESCs. (**A**) A diagram illustrating the genomic locus of miR-290~295 cluster and Nlrp12. Caspase 9 (Cas9) cleavage positions are indicated by red scissors. (**B**) qRT-PCR analysis for mRNA expression of Nlrp12. The β-actin gene was used as a control. For each gene, data were normalized to the mRNA level of wild type ESCs. Shown are mean ± SEM, *n* = 3 biological replicates. The *p*-value was determined by one-way ANOVA followed by two-tailed Tukey’s test. Dg8 KO, Dgcr8 knockout; Dg8/290 DKO, Dgcr8/pri-miR-290~295 double knockout; 290KO, pri-miR-290~295 knockout. (**C**) Volcano plot representation of differential expression analysis of genes in Dgcr8/pri-miR-290~295 double knockout versus Dgcr8 knockout ESCs. The x-axis shows log2 fold changes, and the y-axis shows log10 *p* values. (**D**) Predicted miRNA binding sites in Nlrp12 3′ untranslated region by Targetscan. miRNAs expressed more than 100 copy per cell in wild type ESCs are labeled in red.

We noticed there is a gene Nlrp12 located ~500 base pairs downstream of the miR-290~295 cluster in the opposite direction (Figure 6A). We reasoned that the deletion of the miR-290~295 cluster could dramatically change the transcriptional context of Nlrp12. Indeed, RT-qPCR analysis showed that Nlrp12 was significantly upregulated in Dgcr8/pri-miR-290~295 double knockout versus Dgcr8 knockout ESCs (Figure 6B). To understand the global impact of pri-miR-290~295 knockout, we performed RNA-seq analysis for Dgcr8 knockout and Dgcr8/pri-miR-290~295 double knockout ESCs. There were 89 and 248 genes significantly upregulated or downregulated more than 2 fold (*p*-value < 0.01) upon knockout of pri-miR-290~295. Nevertheless, Nlrp12 was the most significantly altered gene (Figure 6C, Table S2). Moreover, we also confirmed the upregulation of Nlrp12 upon deletion of the miR-290~295 cluster in wild type background ESCs (Figure 6B). Interestingly, the increase of Nlrp12 upon pri-miR-290~295 knockout in wild type background was significantly less than in Dgcr8 knockout background. We hypothesized that the repression by mature miRNAs present in wild type ESCs might explain this difference. Indeed, the 3′ untranslated region of Nlrp12 was predicted to be targeted by multiple miRNAs (Figure 6D), several of which are expressed at relatively high levels (> 100 copy per cell) in wild type ESCs [15]. The deletion of miR-290~295 cluster removed both DNA elements and RNA transcripts; therefore, the upregulation of Nlrp12 could be caused by the loss of either DNA elements or RNA transcripts of pri-miR-290~295. However, since the expression level of pri-miR-290~295 was significantly lower in wild type than Dgcr8 knockout ESCs (Figure 1A,B) due to cleavage by the Microprocessor, and the expression of Nlrp12 was similar in wild type and Dgcr8 knockout ESCs (Figure 6B), it is more likely that the DNA element of pri-miR-290~295 is inhibitory for the transcription of Nlrp12. In other words, the deleted region contained a negative cis-regulatory element for Nlrp12. These results demonstrated that deletion of miRNA genes could have a significant impact on the transcription of neighboring DNA elements. 

**Figure 7:**
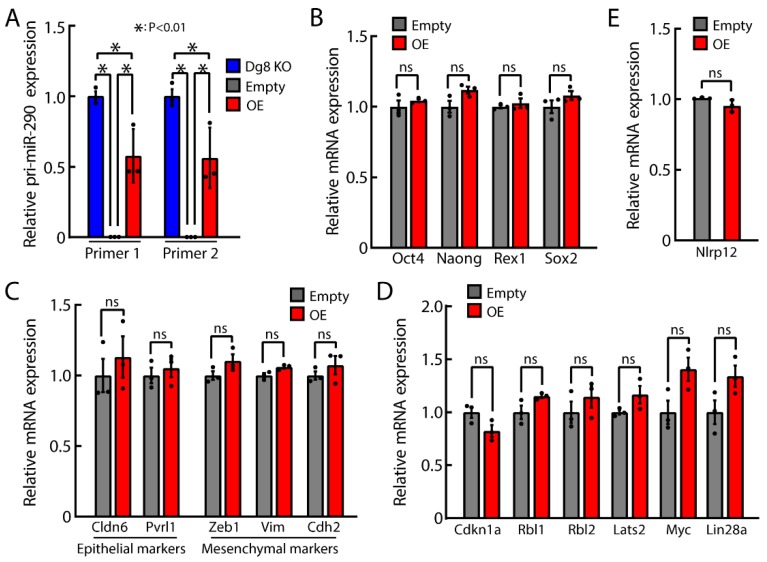
Transgene expression of pri-miR-290~295 has no impact on the expression of pluripotency, EMT- and cell cycle-related genes, and Nlrp12. (**A**) RT-qPCR analysis for pri-miR-290~295 in Dgcr8 knockout, Dgcr8/pri-miR-290~295 knockout ESCs transfected with empty control or vectors overexpressing pri-miR-290~295. The overexpression in double knockout ESCs restored the level of pri-miR-290~295 to ~60% of that in Dgcr8 knockout ESCs. (**B**) RT-qPCR analysis for mRNA expression of pluripotency genes. (**C**) RT-qPCR analysis for mRNA expression of EMT related genes. (**D**) RT-qPCR analysis for mRNA expression of cell cycle-related genes. (**E**) RT-qPCR analysis for mRNA expression of Nlrp12. For all panels, the β-actin gene was used as a control. For each gene, data were normalized to the mRNA level of Dgcr8/pri-miR-290~295 knockout ESCs transfected with empty control except in A, in which data were normalized to Dgcr8 knockout ESCs. Shown are mean ± SEM, *n* = 3 biological replicates. The *p*-value was determined by one-way ANOVA followed by two-tailed Tukey’s test in A and by two-tailed unpaired Student’s *t*-test in Figure 7B–E. Dg8 KO, Dgcr8 knockout; Empty, Dgcr8/pri-miR-290~295 double knockout ESCs transfected with empty control overexpression vectors; OE, Dgcr8/pri-miR-290~295 double knockout ESCs transfected with pri-miR-290~295 overexpression vectors.

To further determine the function of pri-miR-290~295, we overexpressed pri-miR-290~295 transcript in Dgcr8/pri-miR-290~295 double knockout ESCs (Figure 7A). Consistent with results in Figure 2 and Figure 3, overexpression of pri-miR-290~295 did not cause any changes in the expression of pluripotency genes (Figure 7B), EMT-related genes (Figure 7C), or cell cycle genes (Figure 7D). Besides, overexpression of pri-miR-290~295 also did not change the expression of Nlrp12 (Figure 7E), indicating the impact on Nlrp12 by the deletion of pri-miR-290~295 is due to cis-regulation.

## 3. Discussion

Using the Dgcr8 knockout ESC model and miRNA rescue strategy, we and others have shown that mature miR-290~295 miRNAs play important functions in the self-renewal and differentiation of ESCs [8,9,10,11,12,14,16]. However, there remained concerns about accumulated primary miRNA transcripts causing defects in Dgcr8 knockout cells. Here, we generated Dgcr8/pri-miR-290~295 double knockout ESCs. By comparing them with Dgcr8 knockout ESCs, we showed that accumulated pri-miR-290~295 transcripts had no impact on pluripotency, proliferation, G1/S transition, and differentiation. Besides, we found that the knockout of pri-miR-290~295 locus led to dramatic changes in the transcription of neighboring Nlrp12, calling into question the interpretation of miRNA function revealed by genetic deletion of miRNA genes. Interestingly, a previous study [34] showed that the deletion of pri-miR-290~295 led to embryo lethality and defects in germ cells with partial penetrance; whether these defects are actually due to the dysregulation of Nlrp12 but not the loss of miRNAs is worthy of further investigation. Moreover, future research should be designed with sophisticated genetic manipulation tools, such as CRISPR-on and CRISPRi [35,36,37,38,39,40], to reveal molecular mechanisms on how pri-miR-290~295 inhibits the transcription of neighboring genes. 

miRNA biogenesis is a strictly regulated multiple-step process [41]. Mature miRNAs regulate target gene expression by a well-known RNA-induced silencing complex (RISC)-mediated mechanism [42]. Few attentions have been paid on the function of pri- and pre-miRNA beyond miRNA production. However, several reports suggest that pri- and pre-miRNAs encode regulatory information and have a direct function in gene expression, chromatin interaction, and epigenetic state regulation [20,21,22,23]. Trujillo RD et al. showed that pri-miR-let-7 might directly repress target mRNAs, and both loop structure and sequence elements of the pri-miRNA contributed to targeting binding and regulation [21]. A more recent study has identified partially processed RNA intermediates of pri-miR-17~92, which is amplified in numerous cancers and found to downregulate other co-expressed polycistronic miRNAs by competing for the Microprocessor [23]. In a mouse model of cardiac hypertrophy, pri-miR-208b could directly bind to polycomb group protein enhancer of zeste 2 polycomb repressive complex 2 subunit (EZH2) and mediate the silencing of hypertrophic genes [22]. In the current study, we evaluated the function of pri-miR-290~295 in Dgcr8 knockout ESCs. This approach is straightforward for dissecting the function of pri-miRNA transcripts by excluding the interference from the production of mature miRNAs. Knocking out pri-miRNAs in Dgcr8 knockout background may serve as a general approach to initially characterize the function of pri-miRNA transcripts or DNA elements. Unlike Dgcr8 knockout cells, Dicer knockout cells accumulate unprocessed pre-miRNAs [4,5]. Currently, it is not clear whether pre-miRNAs accumulated in Dicer knockout cells have any function on gene expression and cellular phenotypes. Similar strategies, as shown in our study, should be deployed to address the potential influence of pre-miRNAs in Dicer knockout cells in the future.

The miR-290~295 cluster is a major player in regulating multiple functions of ESCs. Studies on the miR-290~295 cluster have great significance in understanding the regulatory networks in the early development of mouse embryos. We have provided the first evidence that accumulated pri-miR-290~295 transcripts have no function in important biological processes of ESCs, including proliferation, cell cycle progression, and pluripotency maintenance. Our results confirmed that Dgcr8 knockout is an ideal strategy at least for studying functions of mature miR-290~295 miRNAs. Nevertheless, our study can not exclude the possibility that accumulated pri-miR-290~295 transcripts may have subtle effects on the expression of certain genes, which could then cause apparent phenotypes at some special circumstances. The impact of pri-miR-290~295 on gene expression should be investigated by global approaches, such as RNA-Seq and proteomics analysis, in the future. These studies will likely reveal general rules on the molecular impact of pri-miRNA transcripts. 

## 4. Materials and Methods

### 4.1. Cell Culture

The mouse ESCs were grown on 0.1% gelatin-coated plates or irradiated mouse embryonic fibroblast (MEF) feeders with ESC culture medium which consists of high glucose DMEM (Hyclone, Singapore, Cat. # 30045.10), 15% fetal bovine serum (PAN, Aidenbach, Bavaria, Germany, Cat. # 2602-P130707), 1000 U/mL mouse leukemia inhibitory factor, 0.1 mM nonessential amino acids (Gibco, Carlsbad, CA, USA, Cat. # 11140050), 1 mM l-glutamine (Gibco, Carlsbad, CA, USA, Cat. # 25030081), 0.1 mM β-mercaptoethanol, penicillin (100 U/mL), and streptomycin (100 μg/mL). To compensate for the proliferation defects of Dgcr8 KO and Dgcr8/miR-290 DKO ESCs, their initial cell numbers were doubled relative to the wild type control in EB and let-7 mediated differentiation experiments. The medium was changed daily, and cells were routinely passaged every other day. For EB differentiation assay, cells were cultured in low-attachment culture dishes in ES medium containing 10% fetal bovine serum and no LIF. At day 8 and day 16, the embryoid bodies were collected for RNA extraction.

### 4.2. Pri-miR-290-295 Cluster Knockout 

Pri-miR-290~295 cluster knockout was performed in the Dgcr8 knockout and wild type ESCs using the CRISPR/Cas9 system. A pair of guide RNAs targeting DNA sequence of the miR-290-295 cluster was designed with the online prediction tool from http://crispr.mit.edu/. The guide RNAs driven by U6 promoter were subcloned into a PiggyBac vector, which contains a Cas9 gene expression cassette and a hygromycin resistance gene cassette. For positive clone screening, around 0.1 million ESCs were plated in 12-well plates, and 1 μg plasmid for each well was transfected using lipofectamine 2000 (Invitrogen, Waltham, MA, USA, Cat.#11668-027), following the manufacturer’s protocol. After 24 h of transfection, the medium was changed with 130 μg/mL Hygromycin B (Roche, Mannheim, Germany, Cat. #10843555001) and then cultured for 4–5 days. Next, surviving cells were harvested and plated at single-cell density on the feeder for single-cell colony isolation. Sequences of gRNAs and PCR primers are listed in Table S3.

### 4.3. Transfection of miRNA Mimics

ESCs in 12-well plate were transfected with 50 nM miRNA mimics (GenePharma, Shanghai, China) using DharmaFECT1 reagent (Dharmacon, Lafayette, CO, USA, Cat. # T-2001) according to the manufacturer’s instruction. A negative control is provided by GenePharma (Shanghai, China), which is a siRNA sequence derived from the *Caenorhabditis elegans* genome that has no targets in the mammalian genome. Seventy-two hours after transfection, the ESCs were collected for further experiments. Sequences of miRNA mimics are shown in Table S4.

### 4.4. RNA Isolation and RT-qPCR

Total RNA was extracted according to standard Trizol protocol (Invitrogen, Waltham, MA, USA, Cat. # 15596026) and was quantified by a Biodropsis BD2000 (OSTC, Beijing, China). Isolated RNA was reverse-transcribed into complementary DNA (cDNA) using the HiScript II QRT SuperMix kit (Vazyme, Nanjing, Jiangsu, China, Cat. # R223). Real-time PCR was performed on Step One Plus Real-Time PCR System (Applied Biosystems, Waltham, MA, USA), and the AceQ qPCR SYBR Green Master Mix kit (Vazyme, Nanjing, Jiangsu, China, Cat. # Q141) was used for gene amplification and quantitation. Sequences of RT-qPCR primers are shown in Table S5.

### 4.5. Alkaline Phosphatase Staining

For AP staining, 1xPBS was used to wash cells followed by 4% paraformaldehyde fixation for 15 min at room temperature. Cells were then stained for alkaline phosphatase using the commercial kit (Vector Laboratories, Burlingame, CA, USA); all the steps were performed according to the manufacturer’s protocol.

### 4.6. Cell Cycle Analysis

For cell cycle analysis, 0.6 million ESCs were plated in 6-well plates and cultured for 24 h. Cells were then gently trypsinized to a single cell suspension and fixed by cold 90% ethanol, then stained with propidium iodide and analyzed by flow cytometry. Cell population doubling time was calculated, as previously described [6].

### 4.7. RNA-Seq and Bioinformatics Analysis

Total RNA was purified by poly-T oligo-attached magnetic beads and then were used to generate double-stranded (ds) cDNA. The ds-cDNA was ligated to adaptors and sequenced by Illumina Genome Analyzer (BerryGenomics, Beijing, China). Sequencing reads were aligned to the mouse genome (mm10) with STAR (version 2.7.0) using the GENCODE transcript annotation as transcriptome guide. All programs were performed under the default settings except for special statements. Expression levels were quantified as normalized FPKM using Cufflinks (version 2.2.1). R 3.5.1 was used for DESeq analysis and the generation of volcano plot. Reads count of pre-miRNAs was normalized to the size of sequencing libraries and presented as reads per million sequencing reads. Pre-miRNAs with fold change >2 in Dgcr8 knockout versus wild type ESCs were taken into account in the next analysis. Accumulated reads count was calculated by subtracting reads count in wild type ESCs from reads count in Dgcr8 knockout ESCs.

### 4.8. Statistical Analysis

Statistical analyses were performed using the GraphPad Prism 8 or Microsoft Excel software. All data in this study were presented as mean ± SEM. We performed one-way ANOVA followed by Tukey’s test for multiple comparisons except in Figure 7B–E, for which we performed two-tailed unpaired Student’s *t*-test. *p*-value < 0.05 was considered as statistically significant.

## Supplementary Materials

Supplementary materials can be found at https://www.mdpi.com/1422-0067/20/18/4345/s1.
